# Are persons with rheumatoid arthritis deconditioned? A review of physical activity and aerobic capacity

**DOI:** 10.1186/1471-2474-13-202

**Published:** 2012-10-18

**Authors:** Tjerk Munsterman, Tim Takken, Harriet Wittink

**Affiliations:** 1 Physical Therapy Center, Martini Hospital Groningen, P.O. Box 30033, 9700 RM, Groningen, Netherlands; 2 School of Clinical Health Sciences, Department of Physical Therapy Science, Utrecht University, Utrecht, the Netherlands; 3 Child Development & Exercise Center, Wilhelmina Children's Hospital, University Medical Center Utrecht, Utrecht, The Netherlands; 4 Research group Lifestyle and Health, Faculty of Health Care, Utrecht University of Applied Sciences, Bolognalaan 101, 3584 CJ, Utrecht, The Netherlands

**Keywords:** Rheumatoid arthritis, Cardiovascular disease, Physical activity, Aerobic capacity, Healthy controls

## Abstract

**Background:**

Although the general assumption is that patients with rheumatoid arthritis (RA) have decreased levels of physical activity, no review has addressed whether this assumption is correct.

**Methods:**

Our objective was to systematically review the literature for physical activity levels and aerobic capacity (VO_2max_). in patients with (RA), compared to healthy controls and a reference population. Studies investigating physical activity, energy expenditure or aerobic capacity in patients with RA were included. Twelve studies met our inclusion criteria.

**Results:**

In one study that used doubly labeled water, the gold standard measure, physical activity energy expenditure of patients with RA was significantly decreased. Five studies examined aerobic capacity. Contradictory evidence was found that patients with RA have lower VO_2max_ than controls, but when compared to normative values, patients scored below the 10^th^ percentile. In general, it appears that patients with RA spend more time in light and moderate activities and less in vigorous activities than controls.

**Conclusion:**

Patients with RA appear to have significantly decreased energy expenditure, very low aerobic capacity compared to normative values and spend less time in vigorous activities than controls.

## Background

Rheumatoid arthritis (RA) is a chronic inflammatory disease characterised by polyarthritis and erosive synovitis and is associated with progressive impairments and activity limitations [1,2]. According to the recent EULAR evidence-based recommendations for cardiovascular risk management in patients with rheumatoid arthritis and other forms of inflammatory arthritis [3], RA should be regarded as a condition associated with higher risk for cardiovascular disease (CVD). The increased risk appears to be due to both an increased prevalence of traditional risk factors and the inflammatory burden of RA [4]. Adequate control of disease activity is necessary to lower the CVD risk and evaluating the effect of lifestyle modification on the CVD risk in inflammatory arthritis was added to the future research agenda [3]. One potentially modifiable lifestyle factor is physical activity (PA). Exercise restrictions, traditionally given to patients with RA because of concerns about aggravating joint inflammation and accelerating joint damage, may contribute to inactivity and deconditioning which is associated with a loss of aerobic capacity [5]. In addition, pain and depression associated with the disease, may result in low PA [6]. Inactivity has been shown to be associated with loss of lean mass, increases in fat mass and metabolic syndrome [6,7], contributing to CVD risk. Several studies do show that physically inactive patients with RA have a significantly worse CVD risk profile compared with physically active patients [8,9]. Conversely, patients with RA with high levels of physical activity (PA) (mean 3342 METhours/week) were shown to have a significantly better CVD risk profile than those with low levels of PA (mean 249 METhours/week), even when adjusting for RA disease duration, activity and severity and steroid use [10]. Recent research also shows that a moderately high or high aerobic capacity, but not high physical activity reduces metabolic syndrome and thus CVD and DM II risk even in obese persons [7].

Little is known about the level of daily PA among persons with RA. For instance, a recent meta-analysis on cardiorespiratory (aerobic) exercise in RA did not examine the influence of baseline physical activity as a confounder on either outcomes or statistical heterogeneity, as this parameter was seldom reported [11]. What is known about PA in patients with RA seems contradictory. Several studies found the proportion of patients with RA meeting national recommendations for PA similar to those of the general population [12-14] or to healthy women the same age [9]. A large international study, however, reported that the majority of patients with RA are inactive [15]. A recent systematic review on physical activity in RA concluded that methodological considerations within the reviewed studies prohibited definitive conclusions on the PA levels in this population [16]. The authors used publications on PA and energy expenditure in patients with RA (N=16) only, including studies that did not use a control group (N=9). The aim of this review did not include gathering evidence on aerobic capacity.

Data on aerobic capacity in patients with RA are hard to find. Aerobic capacity in patients with RA aged 20–65 years was found similar to normative data for the same age groups from a representative sample of the Swedish population [12], but low aerobic capacity is also reported [17]. In this study therefore, we aimed to explore whether individuals with RA are less physically active and experience a decreased aerobic capacity, compared to healthy controls. In addition, we used a healthy reference population [18] to determine the percentile of aerobic capacity for individuals with RA as healthy controls might not be representative of the general population.

## Methods

### Literature search

Electronic databases Medline, Cinahl, Embase, Cochrane and PsycINFO were systematically searched up to November 2010 using the following Mesh terms and text words: (motor activity OR leisure activities OR human activities OR activities of daily living OR aerobic capacity OR energy expenditure) AND rheumatoid arthritis AND healthy, to find studies comparing patients with RA to healthy persons. In order to limit results to adults, the restriction NOT (child OR adolescent) was added. Reference lists from included studies were searched manually for additional relevant studies.

### Inclusion criteria

Studies were included for review when following criteria were met.

the target population: adults with RA (18 years and older)

outcome measures: physical activity, energy expenditure or aerobic capacity

at baseline the outcome measures were compared to those of healthy controls and values and measures of variability were described

### Exclusion criteria

single case reports

studies describing a direct post operative situation

studies written in any language other than English, German or Dutch

Using the above mentioned criteria, a researcher (TM) reviewed the titles of articles in the search printouts from the databases. Abstracts from potentially relevant studies were read and included when all criteria were met. After full text reading articles were finally included when all afore mentioned criteria were met. A manual search of references from included studies was conducted to retrieve further potentially relevant studies.

### Assessing trial characteristics and outcome data

The following information was systematically extracted by reviewers TM and HW: type of study, number of participating patients with RA, sex, age, setting, body composition, disease duration, classification of impairment, use of medicines and outcome measures. Based on consensus, extracted data were included in the review.

### Quality assessment of studies

Although a commonly accepted valid rating instrument concerning the quality assessment of observational studies does not yet exist, there appears to be consensus about important items [19-21]. This review focuses on differences between persons with RA and healthy controls. A comparison of patients and controls at baseline was used in the case of intervention studies. This resulted in the following assessment items: selection of patients and controls, sample size calculation, adjustment for confounding, blinding of assessors and use of statistical analysis.

## Results

**Figure 1:**
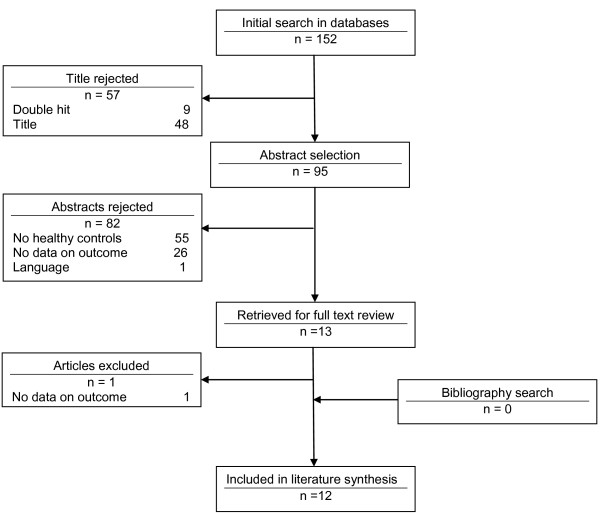
Flow chart literature search.

The literature search yielded 152 studies, from which nine double hits were excluded. Another 48 studies were excluded based on the title. Eighty-two studies were excluded based on abstracts. Thirteen studies were retrieved for full text reading, which resulted in the exclusion of another article because no data concerning afore mentioned outcome measures was given. References tracking of the included studies did not yield new studies. Finally 12 studies were included. Results of the literature search and reasons for exclusion are depicted in Figure 1.

### Quality assessment

The included studies were all observational studies: ten cross-sectional studies and two cohort studies. Six studies included small groups of patients [22-27], sample size varied from 8 to 35 patients. In six other studies [13,14,28-31] sample size ranged from 67 to 232 patients.

In seven studies [14,22,24-27,30] samples of patients and controls were of equal size, in the remaining 5 studies group sizes differed significantly [13,23,28,29,31]. The majority of the patients were recruited from rheumatology or arthritis clinics, in one study from a rehabilitation centre [24] and in two studies [28,29] the recruitment method was unclear. No information on the recruitment of controls was available in six of the studies [22,24-27,29]. In four studies healthy persons living in the same area were recruited [14,22,28,30], in one study healthy relatives acted as controls [31] and one study used information from the general Dutch population as reference [13].

Information about matching of patients and controls was reported in nine studies [22-24,26-31]. Different combinations of the following factors were used: race [26,27], age [22-24,26-28,30], gender [22,24,26,28,30] and body composition [22,26,27]. In one study groups were matched based on genetic and ethnic variables [31]. Having a sedentary lifestyle was a matching criterion in three [22,24,25] of the five studies [22-25,28] investigating aerobic capacity. Physically active participants were included in one study [23], whereas another study included controls to contrast with patients in terms of level of PA and body composition [29].

Three studies reported conducting sample size calculations [13,24,27]. Sample sizes were inadequate with regard to statistical analysis in two cases [13,24] and in the third study [27] adequacy of the sample size was not reported. None of the studies reported blinding of assessors. Six studies [22,24-27,31] reported that data were tested for the assumptions for parametric statistical tests or justified the use of non-parametric tests.

### Patient characteristics

All patients met ACR criteria for RA [32]. Classification into Functional Class (FC) I – IV (mild to severe impairment) [33] was used in seven studies. In two studies patients were classified as FC II [24,28], in five studies patients with different levels of FC were combined in the study population [22,23,25,26,31]. In most studies predominantly female participants were included, with percentages ranging from 62,5% to 88,6%. In three studies women only were included [23,27,31] and in one study gender of participants was not reported [24]. The mean age of patients ranged from 38.1 years to 62.6 years. The use of anti rheumatic drugs and analgesics was described in eight studies [14,23-28,31]. In six studies [14,22,24,25,27,31] patients were excluded because of severe cardio-pulmonary disease, or because they used a walking aid [27].

In three studies [14,24,31] participants with comorbidity other than cardiopulmonary disease were excluded. One study used a body mass index above 30 as an exclusion criterion [25] and exercising regularly was an exclusion criterion in another study [27]. Characteristics of patients and controls, disease parameters and results on aerobic capacity are presented in Table 1. Characteristics of patients and controls, disease parameters and results on EE and PA are presented in Table 2.

**Table 1:** Studies comparing level of aerobic capacity in RA patients and a healthy reference population

**Study (ref nr)**	**n (% F)**	**Reference Population n (% F)**	**Patient characteristics**					**VO**_**2**_**max RA patients versus reference population (ml/kg.min) p: significant difference**	
			**Setting**	**Age [range]**	**Body Composition**	**Disease duration**	**Impairment (FC I-IV)**		
de Carvalho 2003 MT [22]	35 (88.6%)	35♣ (?) hospital staff	Rheumatology clinic	47,85 (8,29)	?	8	FCI: 40% FCII: 51% FCIII: 8.6%	FC I: 24.89 (n=7) FC II: 21.86 (n=6)/24.28 (n=22)	NS
Ekdahl 1992 SMB [28]	67 (62.7%)	77♥ (61.0%) personnel	?	53.0 (10.2)	female: 164.1 (5.6) cm female: 66.1 (11.6) kg male: 177.9 (6.8) cm male: 75.6 (10.7 kg	10.6 (7.8)	FCII	female younger than 54 jr: 22.3±6.8 / 31.7±12.1 older than 54 jr: 18.7±3.5/21.9±5.3 male younger than 54 jr: 24.0±4.3/27.6±7.4 older than 54 jr: 18.7±4.1 / 25.1±6.1	p<0.001 p<0.001 p<0.001 p<0.001
Häkkinnen 2002 MB [23]	23 (100%) ERA LRA	12♠ (100%) area residence	Hospital	ERA: 41 (9) LRA: 49 (7)	ERA: 165 (9) cm 61 (13) kg fat: 30.4 (6.6) % LRA: 164 (7) cm 65 (13) kg fat: 34.3 (7.3) %	ERA: 2.9 (0.6) LRA: 14.5 (4.5)	FCI, II	ERA: 26.7±6.8/LRA: 23.1±6.1/controls: 24.8±2.3	NS
Kurtais 2006 MT [24]	19 (100%)	15♥ (100%) ?	Rehabilitation centre	48.3 (8.4)	?	128.8 (85.6) months	FCI, II	23.7±4.9 / 26.6±6.0	NS
Rall 1996 MB [25]	8 (62.5%)	exercise young : 8 (62.5%) old: 8 (62.5%) control old: 6 (66.7%) ♦	Rheumatology clinic	RA: 41.8±12.6 exercise young : 25.8±2.5 old : 70.3±5.0 control old 68.8±2.9	65.9 (15.9) kg BMI: 25.0 (4.3)	14.6 (12.5)	FC: 2.2 (0.8)	22.9±4.2 / young exercise group: 40.2±10.3 old exercise group: 20.7±5.0 old no exercise group: 21.7±5.7 young exercise versus other groups	p<0.001

Age, body composition, disease duration are expressed as mean (standard deviation) if not expressed otherwise, VO2max is expressed as mean ± standard deviation. Age and disease duration are expressed in years. n: number of participants with RA or in reference population, MB: maximal bicycle test, MT: maximal treadmill test, SMB: sub-maximal bicycle test, %F: percentage females, FC: Functional Class, GP: general population, BMI: body mass index, RA: rheumatoid arthritis, ERA: early RA, LRA: late RA, ml/kg.min: milliliter per kilogram per minute. Controls matched on ♣: sex, age, body mass index, ♠: age, ♥: age, sex,♦: no information on matching.

**Table 2:** Studies comparing level of physical activity in RA patients and a healthy reference population

**Study (ref nr)**	**n (% F)**	**Reference populationn (%F)**	**Patients with RA**					**Physical activity RA patiënts versus reference population p: significant difference**	
			**Setting**	**Age [range]**	**Body Compostion**	**Disease duration**	**Impairment (FC I-IV)**		
v d Berg 2007 [13]	232 (71.1%)	6,428,441* (53%) GP records	Hospital based	62.6 (9.2) 45–64 jr: 58% > 65 jr: 42%	?	?	?	Light: 1297±1009 min/wk / 1495 min/wk Moderate: 369±543 min.wk / 517 min/wk Vigorous: 170±257 min/wk / 187 min/wk Light: 634±795 min/wk / 618 min/wk Moderate: 231±244 min/wk /304 min/wk Vigorous: 250±417 min/wk / 296 min/wk	p=0.01 p=0.01 p=0.01 p=0.01
Lemney 2009 [29]	73 (63.0%)	28♦ (57%) ?	?	52.9 (12.9)	Fat 36.6 (12.8) % p<0.001	?	?	Physical Activity Scale (0–7) 0.8±0.7 / 5.4±1.7	p<0.001
Mancuso 2007 [14]	121 (84%)	120 (91%) Personnel hospital	Hospital based	49 (19–72)	?	14 (10)	?	Walking: 692±610 kcal/w / 1.044±1260 kcal/w Stair climbing: 184±212 kcal/w / 185±262 kcal/w Exercise: 599±848 kcal/w / controls: 729±1210 kcal/w	p=0.002
MacKinnon 2003 [30]	143 (74.8%)	142♥ (72.5%) area residence	Rheumatology clinic	49.7 (11.2)	?	?	?	Work 37.1±19.6 h/wk / 46.5±17.2 h/wk ADL 89.0±15.3 h/wk / 81.1±11.5 h/wk Leisure 38.4±15.4 h/wk / 37.9±15.6 h/wk	p<0.05 p<0.05
Roubenoff 2002 [27]	20 (100%)	20♣ (100%) ?	Arthritis center	47 (14)	BMI 25.3 (4.5)	7.7 (6.5)	FCI FCII	PAEE 2849±1075 kJ/d / 3883±1732 kJ/d PA questionnaire 2188±1075 kJ/d / 3150±1611 kJ/d PA activity monitor 1264±992 kJ/d / 2280±1469 kJ/d	p<0.04 p<0.04 p<0.04
Tourinho 2007 [31]	71 (100)	29♠ (100) sisters/cousins	Rheumatology clinic	38.10 (6.62)	1.57 (0.14) m 62.68 (12.56) kg	30 (7,3)	FC I: 70%	Sedentarism 17.3 % / 3.4% Mild 57.7% / 35% Moderate 25% / 65% Intense 0% / 0% No exercise in leisure time 74% / 66.7%	p=0.004 p=0.004
Roubenoff 1994 [26]	23 (82.6)	23◊ (82.6) ?	Rheumatology clinic	50 (15)	Body cell mass 22.5 (4.3) kg p< 0.000	12.3±8.4 jr	FC I (n=5) FC II (n=10) FC III/IV (n=8)	Vigorous 0.1±0.2 h/d / 1.5±1.5 h/d Moderate 4.0±3.2 h/d / 5.6±2.3 h/d Light 11.6±3.4 h/d / 9.2±3.2 h/d	p<0.0001 p<0.06 p<0.02

Age, body composition,disease duration and physical activity are expresed as mean±standard deviation if not expressed otherwise. n: number of participants with RA or in reference population, RA: rheumatoid arthritis, FC: Functional Class, GP: general population, BMI: body mass index in kilogram per square meter, >: older than.

kcal/wk: kilocalory per week, min/wk: minutes per week, kJ/d: kiloJoule per day, h/d: hours per day. Controls matched on ♣: race, age, body mass index, ♠: genetic factors, ♥: age, sex, ◊: race, age, sex, weight, ♦: controls selected to contrast on body fat, activity level, *: no information on matching.

### Physical activity energy expenditure

PA is defined as: “Any bodily movement produced by skeletal muscles that results in a substantial energy expenditure (EE)” [34]. EE is defined as energy expended during physical activity and measured in calories or joules per unit of time [35]. Physical activity energy expenditure (PAEE) can be calculated using the following equation: PAEE =TEE − REE –TEF, wherein TEE stands for Total EE, REE for resting EE and TEF stands for the thermal effect of food [35]. Three studies that measured REE, by indirect calorimetry, described similar values of REE in patients with RA and healthy controls [25-27], even when results were adjusted for body cell mass and weight [27]. However, when results were adjusted for percentage body fat, REE was higher in persons with RA compared to controls [25,26]. To calculate PAEE TEE was measured, using the doubly labeled water technique (DLW) in one study [27]. In the same study PAEE was estimated using a PA questionnaire and a PA monitor. Using these three forms of measurement PAEE was found to be significantly lower in persons with RA compared to controls. Results from the calculated PAEE correlated with results obtained with the PA monitor (r = 0.37) but not with the PA questionnaire.

### Aerobic capacity

Aerobic capacity was examined in five studies. Two studies conducted a maximal treadmill test [22,24]. A maximal bicycle test was performed in two studies [23,25] and one study used a sub-maximal bicycle test [28].

Both treadmill studies measured oxygen uptake using the breath-by-breath method. Neither study reported significant differences in aerobic capacity between patients and controls. One study used an incremental loading protocol to measure VO_2_ at every stage of the treadmill test [22], until predicted maximal heart rate (HRmax) was reached (220 beats/min – age). Patients and controls were compared at the end of the last stage to determine VO_2max_. However, 50%, 60% and 100% of patients of FC I, II and III respectively, dropped out before reaching the final stage of the test, compared to 5% of the healthy controls. EE at sub-maximal levels was higher in patients of FC II than in the control group, a difference that was not found when comparing patients of FC I with the control group. No statistical test was applied to detect differences between FC III and controls due to the small number of patients of FC III. The other treadmill study [24] used the Bruce protocol. The test was continued until voluntary exhaustion, no criteria for reaching VO_2max_ were given. VO_2max_ did not differ significantly between patients and controls, but this study had inadequate sample size.

Two studies conducted a graded bicycIe test [23,25]. In the first study the test was continued until voluntary exhaustion, however, no criteria for reaching VO_2max_ were given [23]. Patients with early RA (mean disease duration 2.9 years) or long-term RA (mean disease duration 14.5 years) were compared with active healthy peers. At the end of the test mean HR_peak_ of participants fell within 5% of predicted HR_max_ and no significant differences between VO_2 max_ of early RA (ERA), long-term RA (LRA) or control group were found. In the other study [25] VO_2max_ was defined as reaching one of the following three criteria: a plateau in oxygen uptake during the final stage of the test, respiratory exchange ratio > 1.0, or HR_max_ within 10% of expected goal. Patients with RA (mean age 41.8 years) were compared to groups of young and elderly controls. Persons with RA had a lower mean VO_2max_ compared to the young controls (mean age 25.8 years), whereas VO_2max_ of patients and elderly controls (mean age 69.5 years) did not differ significantly.

Finally, VO_2max_ was predicted using a submaximal Åstrand protocol during a bicycle test in one study [28]. Participants were divided in two categories (younger and older than 54 years) and bicycle test results were compared between males or females with RA and healthy controls. Persons with RA had lower estimated VO_2max_ compared to healthy control groups. Females had lower estimated VO_2max_ than males and estimated VO_2max_ decreased with age in all participants.

When comparing VO_2max_ levels of patients with RA to published normative values [18], results showed that patients in all studies scored below the 10^th^ percentile.

### Physical activity level

Six studies used questionnaires to assess PA level, focusing on different aspects of PA [13,14,26,29-31]. Classifying PA based on levels of intensity was done in two studies [29,31]. Using a modified PA Scale to quantify intensity of PA undertaken during a normal week, patients with RA were found to be less engaged in moderate or intense recreational activities or sports [29]. When PA was classified into four levels (sedentary, mild, moderate or intense activities) [31], patients participated on a lower intensity level compared to a reference group. The patients did not engage in regular exercise and were identified to be 15% more sedentary compared to controls. There was a paucity of information on the instrument used to measure PA.

The Paffenbarger questionnaire [26] was used to measure hours per day spent in light, moderate or vigorous forms of PA. PA was defined as walking, stair climbing and sport and converted into EE per week. In another study time spent on PA was recorded during one week using an occupation log consisting of 10 different activities collapsed into 3 categories: work, activities of daily living (ADL) and leisure [30]. Results of these studies [26,30] showed that patients with RA spent an equal amount of time on PA as controls, but more time on light activities [26]. When PA was categorized as ADL, leisure and work, patients spent more time on ADL while spending less on work [30].

Finally the Short Questionnaire to Assess Health Enhancing PA (SQUASH) was used to estimate time spent on light, moderate and vigorous activities in a week [13]. PA was categorized into commuting activities, leisure time activities, household activities, and activities at work and school. Results [32] showed that the proportion of patients with RA meeting the public health recommendation for PA equaled that of the general public (58%), nevertheless younger patients (45 – 65 years) were less active in all categories than controls. Older patients (> 65 years) showed only a significant decrease in moderate PA compared to the reference group.

Modified versions of the Paffenbarger Physical Activity Questionnaire (PPAQ) were used in two trials [14,27] to calculate PAEE. Though similar proportions of patients and controls met recommended minimum levels of EE, patients with RA expended less energy in PA (24.7%), mainly due to walking less (33.7%) [14]. Roubenoff et al. [27] found comparable amounts of mean decreased PAEE in patients with RA (30.5%). Neither studies gave information on the way the PPAQ was modified and the consequences for PA assessment.

## Discussion

The aim of this study was to review the literature about physical activity levels and aerobic capacity in patients with RA, compared to healthy controls and a healthy reference population.

Few studies were available that compared daily PA of persons with RA and healthy control groups. Studies exploring aerobic capacity of persons with RA and healthy controls were even more scarce. Assessment methods, inclusion criteria and methodology of matching controls to patient were heterogeneous, complicating comparisons. No information on the recruitment of controls was available in 50% of the studies and in 25% of the studies there was no information on the matching of patients and controls. Selection bias may threaten the validity of these case–control studies and their results must be interpreted with caution.

### Physical activity energy expenditure

The the gold standard measure to estimate PAEE is doubly labeled water (DLW) [36]. Only one study used DLW to assess EE. Roubenoff et al. [27] combined the use of DLW, a PA monitor and a questionnaire to calculate EE in PA. PAEE and the PA level was significantly lower in patients than controls, while REE was comparable in both groups. EE of patients in this study was comparable to normative data [1]. Results of DLW and the questionnaire did not correlate. This demonstrates a discrepancy between objective and subjective measurement.

### Aerobic capacity

Studies using maximal tests reported no significant differences in aerobic capacity between persons with RA and healthy peers, except for one small sized study (n=8) [25]. Interestingly, in children, adolescents and young adults with Juvenile Arthritis, there is a large body of literature suggesting a reduced VO_2max_ in these patients [37-39].This finding of a comparable aerobic capacity between patients with RA and healthy controls may be due to methodological issues, such as criteria for determining VO_2max_. The use of secondary criteria of RER ≥ 1.10 or 1.15, HR_max_ ± 10 b/min or blood lactate concentration can either lead to a significant undermeasurement of VO_2max_ or reject participants who have actually achieved VO_2max_[40]. The criterium of reaching predicted HR_max_ calculated as 220-age has little scientific basis and can under – or overestimate actual HR_max_ by more than 20 beats/min [41]. In a bicycle study VO_2max_ of patients (mean age 41.8 years) and elderly controls (mean age 69.5 years) did not differ significantly. As VO_2max_ declines with age, this means that patients had a low aerobic capacity. In the study of de Carvalho [22] HR_max_ of patients was significantly lower compared to controls and just reached 90% of predicted HR_max_. In addition, 50%, 60% and 100% of patients of FC I, II and III respectively, dropped out before reaching the final stage of the test, whereas only 5% of healthy controls did, indicating a lower VO_2max_ in RA patients. The other treadmill study of Kurtais et al. [24] had inadequate sample size and did not report if participants reached their HR_max_. In patients with LRA HR_max_ was lower compared to persons with ERA and controls, but was within 10% of predicted HR_max_ in all groups [23]. Rall et al. [25] did not report if participants met preset criteria for reaching VO_2max_.

Small samples combined with large heterogeneity of participants in lifestyle may have compromised the power of these studies [42]. Two studies used sedentary persons as controls [22,24]. Kurtais et al. [24] did not define sedentary, whereas de Carvalho et al. [22] defined sedentary as non-exercising. Hakkinen et al. [23] included physically active controls, but did not define physically active. When aerobic capacity was predicted by conducting a sub-maximal bicycle test [28] patients’ VO_2max_ was decreased compared to controls. At baseline controls exercised more frequently compared to persons with RA. Results may be biased because sub-maximal tests of less fit people tend to underestimate observed values [35]. Moreover, submaximal tests use submaximal HR during exercise, which is influenced by resting HR. It is reported that resting HR is significantly higher in patients with RA [43], but correction for resting HR was not reported. Minor et al. [44,45] also showed that the correlation of predicted VO_2max_ and actual VO_2max_ diminished from 0.96 to 0.77 when a sub-maximal treadmill test, validated for use in a healthy population, was used for testing patients with RA.

Some reviewed studies included patients according to functional class. In the study that described results for separate functional classes [22], VO_2max_ decreased from FCI to FCIII and sub-maximal energy expenditure was higher in FCII and FCIII compared to FCI and controls. When VO_2max_ of persons with ERA and LRA was compared, persons with ERA experienced less disability and had higher VO_2max_ than persons with LRA and controls [23]. Although no information was available it is plausible that more persons of higher FC were allocated in de LRA group.

When comparing patients with RA VO_2max_ values to published normative data on healthy adults, patients consistently scored below the 10^th^ percentile, indicating severe loss of aerobic capacity.

Studies with large, unselected, samples of patients with RA using rigorous exercise testing protocols and methodology are warranted [18].

### Physical activity level

We found 6 studies, using 6 different questionnaires, limiting comparison across studies. In general it appears that patients with RA spend more time in light and moderate activities and less in vigorous activities than controls. Patients in the trial of de Caravalho et al. [22] expended significantly more energy than healthy controls, up to 17,9%, especially at normal walking speeds (3–5 km/h) [22]. Questionnaires using a standard intensity categorization of PA might therefore underestimate energy expenditure of patients with RA. Some researchers report that one third of persons with RA experience cachexia [26,46], defined as a loss of muscle mass combined with higher fat mass and higher REE. Matching persons with RA and healthy controls on BMI and comparing EE based on METs may therefore lead to a biased result [47].

McKinnon et al. [30] found that patients were 20% less physically active, caused by less time spent on work. This may be due to the fact that more patients were unemployed compared to healthy controls: 43% - 86%, respectively [30]. Tourinho et al. [31] reported similar differences: 17.3% - 96.7% Patients with RA are at risk to become unemployed with increasing age and disease duration [48]. When PA level of persons with RA was compared to data from the Dutch population patients were less active. However, employed patients and controls showed comparable levels of PA (2577 min/wk vs 2433 min/wk) [13]. Mancuso et al. [14] found a decreased EE of 24.7% in patients compared to controls, even though all participants were employed at the start of the trial. Differences in results compared to other studies may have occurred because time spent at work is not assessed in the Paffenbarger Index.

Reduced vigorous activities and low VO_2max_ in patients with RA will place these patients at even greater risk for the metabolic syndrome and thus cardiovascular disease. We recommend counseling these patients on the benefits of exercise and doing research on the effect of (increased) exercise on CVD risk. Further research into factors that predict higher levels of PA in this population is to be recommended.

## Conclusion

According to the gold standard persons with RA were less active compared to healthy subjects. The results of this review cannot support or refute a conclusion that persons with RA have decreased aerobic capacity compared to healthy controls. This may reflect the fact that half the general population is inactive. However, when compared to normative values patients as well as controls had very low percentile rank of aerobic capacity.

In general it appears that patients with RA spend more time in light and moderate activities and less in vigorous activities than controls. A higher functional class seems to be related to higher sub-maximal EE and lower levels of PA and aerobic capacity. Unemployment, possibly related to RA impairment, seems to contribute to a diminished physical activity level. Patients with RA suffer a 60% greater risk of CVD. Whether this risk can be reduced by a moderately high or high aerobic capacity as in healthy subjects, should be a research priority. Improving aerobic capacity in patients with RA seems of the highest importance and vigorous physical activity can contribute to this. Aerobic training in stable RA has been shown to be safe [11] and improve aerobic capacity [49]. Future research should employ rigorous exercise testing protocols and methodology including the use of criteria for reaching VO_2max,_ differentiate between patients of different functional classes and take employment status into account.

## Competing interests

The authors have declared no conflicts of interest.

## Authors’ contributions

TM: made substantial contributions to conception and design, acquisition of data, analysis and interpretation of data and drafting the manuscript. TT: revised the paper critically for important intellectual content; and gave final approval of the version to be published. HW: made substantial contributions to conception and design, analysis and interpretation of data, revised the paper critically for important intellectual content; and gave final approval of the version to be published. All authors read and approved the final manuscript.

## Pre-publication history

The pre-publication history for this paper can be accessed here:

http://www.biomedcentral.com/1471-2474/13/202/prepub

## References

[B1] Plasqui G The role of physical activity in rheumatoid arthritis Physiol Behav 2008 94 270 275 10.1016/j.physbeh.2007.12.012 18234247

[B2] van der Heijde D Impact of rheumatoid arthritis on physical function during the first five years. No longer a question mark? Rheumatology (UK) 2000 39 579 580 10.1093/rheumatology/39.6.579 10888700

[B3] Peters MJ Symmons DP McCarey D Dijkmans BA Nicola P Kvien TK EULAR evidence-based recommendations for cardiovascular risk management in patients with rheumatoid arthritis and other forms of inflammatory arthritis Ann Rheum Dis 2010 69 325 331 10.1136/ard.2009.113696 19773290

[B4] Turesson C Jacobsson LT Matteson EL Cardiovascular co-morbidity in rheumatic diseases Vasc Health Risk Manag 2008 4 605 614 18827910 10.2147/vhrm.s2453 PMC2515420

[B5] Mayoux-Benhamou A Giraudet-Le Quintrec JS Ravaud P Champion K Dernis E Zerkak D Influence of patient education on exercise compliance in rheumatoid arthritis: a prospective 12-month randomized controlled trial J Rheumatol 2008 35 216 223 18085742

[B6] Giles JT Bartlett SJ Andersen RE Fontaine KR Bathon JM Association of body composition with disability in rheumatoid arthritis: impact of appendicular fat and lean tissue mass Arthritis Rheum 2008 59 1407 1415 10.1002/art.24109 18821641 PMC2670990

[B7] Elkan AC Hakansson N Frostegard J Cederholm T Hafstrom I Rheumatoid cachexia is associated with dyslipidemia and low levels of atheroprotective natural antibodies against phosphorylcholine but not with dietary fat in patients with rheumatoid arthritis: a cross-sectional study Arthritis Res Ther 2009 11 R37 10.1186/ar2643 19284557 PMC2688183

[B8] Metsios GS Stavropoulos-Kalinoglou A Sandoo A van Zanten JJ Toms TE John H Vascular function and inflammation in rheumatoid arthritis: the role of physical activity Open Cardiovasc Med J 2010 4 89 96 20361002 10.2174/1874192401004020089 PMC2847820

[B9] Elkan AC Hakansson N Frostegard J Hafstrom I Low level of physical activity in women with rheumatoid arthritis is associated with cardiovascular risk factors but not with body fat mass - a cross sectional study BMC Musculoskelet Disord 2011 12 13 10.1186/1471-2474-12-13 21235741 PMC3027198

[B10] Metsios GS Stavropoulos-Kalinoglou A Panoulas VF Wilson M Nevill AM Koutedakis Y Association of physical inactivity with increased cardiovascular risk in patients with rheumatoid arthritis Eur J Cardiovasc Prev Rehabil 2009 16 188 194 10.1097/HJR.0b013e3283271ceb 19238083

[B11] Baillet A Zeboulon N Gossec L Combescure C Bodin LA Juvin R Efficacy of cardiorespiratory aerobic exercise in rheumatoid arthritis: meta-analysis of randomized controlled trials Arthritis Care Res (Hoboken ) 2010 62 984 992 10.1002/acr.20146 20589690

[B12] Eurenius E Stenstrom CH Physical activity, physical fitness, and general health perception among individuals with rheumatoid arthritis Arthritis Rheum 2005 53 48 55 10.1002/art.20924 15696555

[B13] van den Berg MH de Boer I le CS Breedveld FC Vliet Vlieland TP Are patients with rheumatoid arthritis less physically active than the general population? J Clin Rheumatol 2007 13 181 186 10.1097/RHU.0b013e318124a8c4 17762450

[B14] Mancuso CA Rincon M Sayles W Paget SA Comparison of energy expenditure from lifestyle physical activities between patients with rheumatoid arthritis and healthy controls Arthritis Rheum 2007 57 672 678 10.1002/art.22689 17471544

[B15] Sokka T Hakkinen A Kautiainen H Maillefert JF Toloza S Mork HT Physical inactivity in patients with rheumatoid arthritis: data from twenty-one countries in a cross-sectional, international study Arthritis Rheum 2008 59 42 50 10.1002/art.23255 18163412

[B16] Tierney M Fraser A Kennedy N Physical Activity in Rheumatoid Arthritis: A Systematic Review J Phys Act Health 2011 10.1123/jpah.9.7.1036 22971883

[B17] Chang CL Chiu CM Hung SY Lee SH Lee CS Huang CM The relationship between quality of life and aerobic fitness in patients with rheumatoid arthritis Clin Rheumatol 2009 28 685 691 10.1007/s10067-009-1132-0 19340515

[B18] Gibbons RJ Balady GJ Beasley JW FAAFP JW Bricker JT Duvernoy WFC ACC/AHA Guidelines for Exercise Testing: Executive Summary: A Report of the American College of Cardiology/ American Heart Association Task Force on Practice Guidelines (Committee on Exercise Testing) Circulation 1997 96 345 354 10.1161/01.CIR.96.1.345 9236456

[B19] Deeks JJ Dinnes J D'Amico R Sowden AJ Sakarovitch C Song F Evaluating non-randomised intervention studies Health Technol Assess 2003 7 10.3310/hta7270 14499048

[B20] Mallen C Peat G Croft P Quality assessment of observational studies is not commonplace in systematic reviews J Clin Epidemiol 2006 59 765 769 10.1016/j.jclinepi.2005.12.010 16828667

[B21] West S King V Carey TS Lohr KN McKoy N Sutton SF Systems to rate the strength of scientific evidence Evid Rep Technol Assess (Summ ) 2002 1 11 11979732 PMC4781591

[B22] de Carvalho MR Tebexreni AS Salles CA Barros NT Natour J Oxygen uptake during walking in patients with rheumatoid arthritis–a controlled study J Rheumatol 2004 31 655 662 15088289

[B23] Hakkinen A Haanonan P Nyman K Hakkinen K Aerobic and neuromuscular performance capacity of physically active females with early or long-term rheumatoid arthritis compared to matched healthy women Scand J Rheumatol 2002 31 345 350 10.1080/030097402320817068 12492249

[B24] Kurtais Y Tur BS Elhan AH Erdogan MF Yalcin P Hypothalamic-pituitary-adrenal hormonal responses to exercise stress test in patients with rheumatoid arthritis compared to healthy controls J Rheumatol 2006 33 1530 1537 16881110

[B25] Rall LC Meydani SN Kehayias JJ wson-Hughes B Roubenoff R The effect of progressive resistance training in rheumatoid arthritis. Increased strength without changes in energy balance or body composition Arthritis Rheum 1996 39 415 426 10.1002/art.1780390309 8607890

[B26] Roubenoff R Roubenoff RA Cannon JG Kehayias JJ Zhuang H wson-Hughes B Rheumatoid cachexia: cytokine-driven hypermetabolism accompanying reduced body cell mass in chronic inflammation J Clin Invest 1994 93 2379 2386 10.1172/JCI117244 8200971 PMC294444

[B27] Roubenoff R Walsmith J Lundgren N Snydman L Dolnikowski GJ Roberts S Low physical activity reduces total energy expenditure in women with rheumatoid arthritis: implications for dietary intake recommendations American Journal of Clinical Nutrition 2002 76 774 779 12324290 10.1093/ajcn/76.4.774

[B28] Ekdahl C Broman G Muscle strength, endurance, and aerobic capacity in rheumatoid arthritis: a comparative study with healthy subjects Ann Rheum Dis 1992 51 35 40 10.1136/ard.51.1.35 1540034 PMC1004615

[B29] Lemmey A Maddison P Breslin A Cassar P Hasso N McCann R Association between insulin-like growth factor status and physical activity levels in rheumatoid arthritis J Rheumatol 2001 28 29 34 11196538

[B30] MacKinnon JR Miller WC Rheumatoid Arthritis and Self Esteem: The Impact of Quality Occupation J Occupational Sc 2003 10 90 98 10.1080/14427591.2003.9686515

[B31] Tourinho TF Capp E Brenol JC Stein A Physical activity prevents bone loss in premenopausal women with rheumatoid arthritis: A cohort study Rheumatol Int 2008 28 1001 1007 10.1007/s00296-008-0554-3 18317768

[B32] Arnett FC Hunder GG The American Rheumatism Associatio 1987 revised criteria for the classification of rheumatoid arthritis Arthritis & Rheum 1988 31 315 324 10.1002/art.1780310302 3358796

[B33] Hochberg MC Chang RW Dwosh I Lindsey S Pincus T Wolfe F The American College of Rheumatology 1991 revised criteria for the classification of global functional status in rheumatoid arthritis Arthritis Rheum 1992 35 498 502 10.1002/art.1780350502 1575785

[B34] Caspersen CJ Powell KE Christenson GM Physical activity, exercise, and physical fitness: definitions and distinctions for health-related research Public Health Rep 1985 100 126 131 3920711 PMC1424733

[B35] McArdle WD Katch FI Katch VL Exercise physiology, sixth edition edn 2007 Williams & Wilkins, Lippincott

[B36] Pols MA Peeters PH Kemper HC Grobbee DE Methodological aspects of physical activity assessment in epidemiological studies Eur J Epidemiol 1998 14 63 70 10.1023/A:1007427831179 9517875

[B37] Takken T Hemel A van der NJ Helders PJ Aerobic fitness in children with juvenile idiopathic arthritis: a systematic review J Rheumatol 2002 29 2643 2647 12465166

[B38] Lelieveld OT van BM Takken T van WE Van Leeuwen MA Armbrust W Aerobic and anaerobic exercise capacity in adolescents with juvenile idiopathic arthritis Arthritis Rheum 2007 57 898 904 10.1002/art.22897 17665473

[B39] van Brussel M Lelieveld OT van der Net J Engelbert RH Helders PJ Takken T Aerobic and anaerobic exercise capacity in children with juvenile idiopathic arthritis Arthritis Rheum 2007 57 891 897 10.1002/art.22893 17665476

[B40] Poole DC Wilkerson DP Jones AM Validity of criteria for establishing maximal O2 uptake during ramp exercise tests Eur J Appl Physiol 2008 102 403 410 10.1007/s00421-007-0596-3 17968581

[B41] Robergs RA Landwehr R The surprising history of the "HRmax=220-age" equation JEPonline 2002 5

[B42] Portney LG Watkins MPfoundations of clinical research, applications to practice, second edition edn 2000 Health, Prentice Hall

[B43] Piha SJ Voipio-Pulkki LM Elevated resting heart rate in rheumatoid arthritis: possible role of physical deconditioning Br J Rheumatol 1993 32 212 215 10.1093/rheumatology/32.3.212 8448611

[B44] Ebbeling CB Ward A Puleo EM Widrick J Rippe JM Development of a single-stage submaximal treadmill walking test Med Sci Sports Exerc 1991 23 966 973 1956273

[B45] Minor MA Johnson JC Reliability and validity of a submaximal treadmill test to estimate aerobic capacity in women with rheumatic disease J Rheumatol 1996 23 1517 1523 8877918

[B46] Metsios GS Stavropoulos-Kalinoglou A Douglas KM Koutedakis Y Nevill AM Panoulas VF Blockade of tumour necrosis factor-alpha in rheumatoid arthritis: effects on components of rheumatoid cachexia Rheumatology (Oxford) 2007 46 1824 1827 10.1093/rheumatology/kem291 18032540

[B47] Byrne NM Hills AP Hunter GR Weinsier RL Schutz Y Metabolic equivalent: one size does not fit all J Appl Physiol 2005 99 1112 1119 10.1152/japplphysiol.00023.2004 15831804

[B48] Geuskens GA Burdorf A Hazes JM Consequences of rheumatoid arthritis for performance of social roles–a literature review J Rheumatol 2007 34 1248 1260 17407220

[B49] Hurkmans E van der Giesen FJ Vliet Vlieland TP Schoones J Van den Ende EC Dynamic exercise programs (aerobic capacity and/or muscle strength training) in patients with rheumatoid arthritis Cochrane Database Syst Rev 2009 CD006853 19821388 10.1002/14651858.CD006853.pub2 PMC6769170

